# Potential of real-time pollen data: perceived needs and symptom management among seasonal allergic rhinitis patients

**DOI:** 10.3389/falgy.2026.1850404

**Published:** 2026-06-02

**Authors:** Nora van Gaal, Christine Boomsma, Lisbeth F. Hall, Jannie J. van der Helm

**Affiliations:** 1 Centre for Environmental Safety and Security, National Institute for Public Health and the Environment (RIVM), Bilthoven, Netherlands; 2 Netherlands School of Public and Occupational Health (NSPOH), Utrecht, Netherlands

**Keywords:** allergen avoidance, automatic monitoring, informational needs, real-time pollen information, seasonal allergic rhinitis

## Abstract

**Introduction:**

Seasonal allergic rhinitis (SAR) affects up to 20% of the Dutch population and significantly impacts daily functioning and quality of life. Despite behavioural measures and pharmacotherapy, many individuals experience suboptimal symptom control. Real-time pollen data are increasingly available and may support improved anticipation and management of symptoms. We examined its perceived value and intended use.

**Methods:**

A cross-sectional online questionnaire was conducted from April to June 2024 among 961 adults with self-reported or physician-diagnosed SAR, recruited via social media of the Dutch National Institute for Public Health and the Environment (RIVM). The questionnaire assessed symptoms, management strategies and effectiveness, perceived informational needs and usage, and demographic variables. Multinomial logistic regression identified factors associated with perceived informational needs.

**Results:**

Most participants used behavioural measures (58.1%) and medication (92.1%) to manage SAR symptoms, though perceived effectiveness varied. One in four expressed a need for more symptom management information. Younger age and moderate perceived impact on daily life were associated with increased informational needs on managing SAR. A third (35.4%) thought that real-time pollen data could contribute to managing SAR, with male gender and university education being associated with a perceived possible contribution. Real-time pollen data was valued for: confirmation of symptoms, limiting exposure to pollen and adjusting medication.

**Discussion:**

Findings indicate the potential for further optimisation of SAR symptom management and for fulfilling informational needs. Particularly men and those with university education thought that real-time pollen data could contribute to managing SAR. Information on the possible preventive use of pollen forecasts via accessible digital tools could enhance SAR self-management.

## 1 Introduction

Seasonal allergic rhinitis (SAR)—commonly known as hay fever or pollen allergy—is a chronic condition. Pollen are common aeroallergens responsible for symptoms such as dyspnea, coughing, rhinorrhea, conjunctival irritation, and general malaise (1). The symptoms can have major impacts on daily functioning, and the health and economic burden of disease is considerable (2–4). The overall prevalence of SAR in the Netherlands is currently approximately 15%–20%, compared to prevalence estimates of up to 40% across Europe (5, 6). Worldwide SAR prevalence has been increasing in recent decades (7, 8). Moreover, the timing, intensity, and geographic distribution of pollen in Europe is changing due to factors such as climate change, increased international travel, and urban landscaping (6, 9).

Proper and timely allergy management can relieve symptoms and consequently minimizes the burden of disease (10). Management guidelines for SAR recommend a stepwise approach, including allergen avoidance behaviours (e.g., staying indoors, wearing sunglasses), pharmacotherapy (e.g., antihistamines, corticosteroids), and allergen immunotherapy (11). Allergen avoidance can reduce or eliminate SAR symptoms and the amount of pharmacotherapy needed for symptom control (12). Pharmacotherapy offers effective symptom relief, particularly when used preventively and continuously (13). However, many people are not able to manage their symptoms optimally despite the use of pharmacotherapy, with up to two-thirds of patients continuing to experience symptoms (14–16). One way to help people improve SAR symptom management is through airborne pollen monitoring, forecasting and alerting.

Since the late 1960s, airborne pollen monitoring has enabled researchers to track seasonal variations in allergen exposure (17). It is typically conducted manually through microscopic analysis and counting of pollen samples on Hirst-type volumetric traps, introducing time lags of several days to a week (17). Recent advances in technology have led to automated pollen sampling and analysis, providing real-time information about temporal and spatial variations during the flowering seasons of allergenic plants, and forming a basis for more accurate pollen forecasting (17–19). Pollen forecasting and alerting could empower individuals to better anticipate symptom flare-ups by adjusting daily routines, and optimizing the timing of pharmacotherapy (20). While real-time pollen information is increasingly available, it is still insufficiently understood which patients express a need for it, how they expect to use it, and what personal or clinical characteristics are associated with that need. We studied how patients with seasonal allergic rhinitis (SAR) in the Netherlands perceive the effectiveness of their current self-reported symptom management strategies. Furthermore, we examined additional informational needs regarding these strategies and the use of real-time pollen data. Also, we assessed how patients perceive the possible contribution of real-time pollen data and factors associated with this perception. The findings are intended to inform the development of tailored, equitable, digital tools that support self-management of SAR.

## 2 Materials and methods

### 2.1 Study design and participants

A cross-sectional online questionnaire was conducted using the Qualtrics platform between April 29 and June 4, 2024. The questionnaire was distributed via the social media channels (Facebook, LinkedIn, Instagram, and X) of the Dutch National Institute for Public Health and the Environment (RIVM). The study was promoted through posts on these platforms, using general public-facing channels. Posts were shared multiple times during the recruitment period to maximize reach. Participants accessed the questionnaire via a direct link in these posts. These posts explained that the study aimed to gain insight into the experiences and informational needs of people with hay fever. Recruitment relied on voluntary participation, with individuals encountering the study either because they followed RIVM channels or because the posts were shared within their networks. Eligibility criteria included age ≥18 years and a self-reported or physician-diagnosed history of SAR; no clinical verification was performed.

### 2.2 Questionnaire

The questionnaire comprised five sections: (1) SAR symptoms and burden (diagnosis, symptom type and severity, impact on daily life); (2) symptom management (allergy medication and behavioural allergen avoidance measures); (3) perceived informational needs related to disease understanding and prevention; (4) use and perceived possible contribution of real-time pollen information and alerts (“real-time” refers to pollen data updated hourly) on managing SAR; and (5) demographics (age, gender, education, and employment).

### 2.3 Patient and public involvement

The questionnaire was developed in consultation with experts from Leiden University Medical Center, Elkerliek Ziekenhuis Helmond, Technical University Munich and Reinier de Graaf Gasthuis Delft, and it was refined based on two focus group sessions and a pilot questionnaire including 27 individuals with SAR.

### 2.4 Ethical considerations

Participation was voluntary, and informed consent was obtained at the start of the questionnaire. All data was collected anonymously and handled in accordance with Dutch privacy legislation and the Declaration of Helsinki.

### 2.5 Data analysis

The questionnaire platform required all questions to be answered, resulting in a complete dataset. Data were analysed using IBM SPSS Statistics, version 29.0.1.0 (171). Descriptive statistics were used to summarize characteristics of the participants and key questionnaire responses. To identify factors that were associated with the perceived need for additional information and with the perceived possible contribution of real-time pollen data on managing SAR, multinomial logistic regression analyses were performed. The outcome variable had three categories: “Not sure” was used as the reference category, to analyse which characteristics positively (“Yes”) or negatively (“No”) related to the reported perceived need for information. Factors analysed were: age, gender, education level, allergy test performed, pulmonary co-diagnosis (yes/no), impact on daily life, medication use and/or behavioural measures. We checked for linearity and multicollinearity, and the assumptions were met. The model fit was assessed using the Nagelkerke *R*^2^. A *p*-value of <0.05 was considered statistically significant.

## 3 Results

A total of 961 participants completed the questionnaire. Most participants were female (76.7%) and the median age was 42.0 years (IQR: 33–52). Most had higher or university level education (69.1%). Nearly half (47.6%) had a confirmed allergy. A pulmonary co-diagnosis was reported by 221 (23.0%); a total of 15.8% had an asthma diagnosis (Table 1).

**Table 1:** Epidemiological characteristics of the study population in The Netherlands, 2024.

Characteristic	*N*	%
Total	961	100%
Gender		
Male	218	22.7%
Female	737	76.7%
Other	2	0.2%
Prefer not to say	4	0.4%
Median age in years (IQR)	42.0 (33–52)	
Education level		
No education	2	0.2%
Primary education	6	0.6%
Secondary education	73	7.6%
Intermediate education	195	20.3%
Higher education	339	35.3%
University education	325	33.8%
Prefer not to say	21	2.2%
Allergy test performed		
No	504	52.4%
Allergy confirmed	457	47.6%
Pulmonary co-diagnosis		
None	740	77.0%
Asthma	152	15.8%
COPD	11	1.1%
Other	58	6.0%

Table 2 summarizes symptom burden, management strategies, and perceived informational needs.

**Table 2:** Symptom burden, management, and informational needs of the study population in The Netherlands, 2024 (*N* = 961).

	*N*	%
Severity of the symptoms		
Mild	44	4.6%
Fairly mild	118	12.3%
Not mild/not severe	398	41.4%
Fairly severe	335	34.9%
Severe	66	6.9%
Impact on daily life		
Not at all	32	3.3%
Hardly	277	28.8%
Moderate extent	496	51.6%
Great extent	114	11.9%
Very great extent	42	4.4%
Period during which symptoms were experienced		
Throughout the entire year	159	16.5%
Continuously during a specific period of the year	547	56.9%
Occasionally during a specific period of the year	218	22.7%
Other	37	3.9%
Months in 2023 during which symptoms were experienced*		
January	62	6.5%
February	228	23.7%
March	472	49.1%
April	644	67.0%
May	715	74.4%
June	599	62.3%
July	474	49.3%
August	398	41.4%
September	301	31.3%
October	107	11.1%
November	28	2.9%
December	28	2.9%
Change in symptoms over the past 5 years*		
Symptoms have worsened	453	47.1%
Symptoms started earlier in the calendar year	423	44.0%
Symptoms lasted longer in the calendar year	223	23.2%
Symptoms have lessened	120	12.5%
No change in symptoms	156	16.2%
Medication use		
None	76	7.9%
1 medicine, prescribed	184	19.1%
1 medicine, over-the-counter	212	22.1%
Multiple, prescribed	287	29.9%
Multiple, over-the-counter	86	8.9%
Multiple, prescribed/over-the-counter	116	12.1%
Period of medication use		
Throughout the year	196	20.4%
Consistently during a certain period	443	46.1%
Intermittently during a certain period	212	22.1%
Otherwise	34	3.5%
No medication	76	7.9%
Behavioural measures		
Yes, one or multiple	558	58.1%
None	403	41.9%
Types of behavioural measures		
Drying the washing inside	252	26.2%
Not exercising stenuously outside	147	15.3%
Rinsing hair before going to bed	134	13.9%
Avoiding areas with high pollen concentrations	104	10.8%
Keeping track of reports on the amount and types of pollen in the air	234	24.3%
Installing window screens with pollen filters	111	11.6%
Keeping windows and doors closed as much as possible	271	28.2%
Filtering indoor air with an air purifier	135	14.0%
Wearing a face mask outdoors	11	1.1%
Applying petroleum jelly inside the nose	199	20.7%
Other self-reported measures (e.g., wearing sunglasses, acupuncture, using tape from physiotherapist and eating honey from the region)	178	18.5%
Medication use and/or behavioural measures		
Medication only	358	37.3
Behavioural measures only	31	3.2%
Medication use and behavioural measures	527	54.8%
No use of medication or behavioural measures	45	4.7%
Perceived need for additional information on managing allergic rhinitis symptoms		
Yes	238	24.8%
No	397	41.3%
Not sure	326	33.9%
Perceived possible contribution of real-time pollen information on managing allergic rhinitis symptoms		
Yes	340	35.4%
No	251	26.1%
Not sure	370	38.5%
Expected benefits of using real-time pollen information^1^^,^^*^		
Preventing symptoms by avoiding pollen	155	21.8%
Alleviating symptoms by limiting exposure to pollen	362	51.0%
Adjusting medication	350	49.3%
Confirmation of allergy symptoms	382	53.8%
Expectation that real-time pollen alerts can contribute to managing allergic rhinitis symptoms		
Yes	466	48.5%
No	285	29.7%
Not sure	210	21.9%
Preferred mode of access to real-time pollen alerts^2^^,^^*^		
Website/app	536	79.3%
Email	78	11.5%
SMS	161	23.8%
No preference/not sure	74	10.9%
Other	15	2.2%

* Results for this question are non-cumulative.

1 Question only for the participants who answered “yes” or “not sure” to the previous question: “perceived possible contribution of real-time pollen information on managing allergic rhinitis symptoms” (*N* = 710).

2 Question only for the participants who answered “yes” or “not sure” to the previous question: “expectation that real-time pollen alerts can contribute to managing allergic rhinitis symptoms” (*N* = 676).

### 3.1 Symptom burden

Moderate to fairly severe symptoms were common (76.3%), with 47.1% reporting worsening SAR over the past five years. Earlier onset (44.0%) and longer duration (23.2%) of symptoms were frequently noted. A moderate impact of SAR on daily life was reported by 51.6%, while 16.3% experienced a large or very large impact.

### 3.2 Perceived effectiveness of medication on alleviating allergy symptoms

Prescribed and/or over-the-counter medication use was high (92.1%), with 29.9% using multiple prescriptions. To prevent or alleviate allergy symptoms, 22.0% used medication throughout the year, 50.1% used it consistently during a certain period and 24.0% used it intermittently during a certain period.

The perceived effectiveness of allergy medication by participants varied considerably. The majority rated their medication as either moderately effective (51.7%) or effective to a large or very large extent (32.5%). A smaller proportion indicated that their medication was hardly effective (14.8%) or not effective at all (1.0%). The perceived effectiveness of the medication did not differ for the period it was used (throughout the entire year, continuously during a specific period of the year, occasionally during a specific period of the year, *χ*^2^(4) = 5.36, *p* = 0.253) nor for the type of medication used (prescribed, over-the-counter or both, *χ*^2^(4) = 8.61, *p* = 0.072). Perceived effectiveness also did not differ for participants using multiple medications (either prescribed, over-the-counter, or both) compared to those using a single medication [*χ*^2^(2) = 4.79, *p* = 0.091].

### 3.3 Perceived effectiveness of behavioural measures on alleviating allergy symptoms

Out of the total study population (*N* = 961) most participants (54.8%) used a combination of medication and behavioural measures, 37.3% used medication only and 3.1% used behavioural measures only. Behavioural measures were used by 558 participants (58.1%). Over half of these participants (55.2%) indicated that such measures were moderately effective, while 32.6% rated them as hardly effective. Smaller proportions reported the measures as not effective at all (1.1%), effective to a large extent (8.8%), or to a very large extent (2.3%). The most frequently used measures encompassed drying the washing inside, tracking the amount and types of pollen in the air, and keeping windows and doors closed as much as possible.

### 3.4 Perceived need for additional information on managing allergic rhinitis symptoms

The majority of participants (58.7%) indicated that they had an additional need for information on managing symptoms or were not sure about an additional need for information. Out of these 564 participants, most indicated a need for information on what interventions can be taken to minimize symptoms (86.7%), followed by information on available medication for managing SAR (76.6%), on minimizing or preventing exposure (73.9%), and on SAR in general (46.1%).

A multinomial logistic regression analysis (Table 3) was conducted to examine whether socio-demographic factors (age, gender, education) and SAR symptom (management) factors (allergy testing, pulmonary diagnosis, medication use, behavioural measures, impact on daily life) are associated with a perceived need for additional information. The overall model was significant, indicating that the set of variables reliably distinguished between response categories [*χ*^2^(26) = 56.2, *p* < 0.001]. But the explanatory power was relatively low (Nagelkerke *R*^2^ = 0.07), indicating that informational needs are influenced by additional, unmeasured factors. Two factors were shown to be associated with perceived need for additional information. Younger participants (<35 years) were more likely to report a need for information (“Yes” vs. “Not sure”) [OR = 2.37 95% CI (1.33, 4.24)] compared to participants >=55 years old. Those who reported a moderate impact on daily life were also more likely to report a need for information (“Yes” vs. “Not sure”) [OR = 1.73, 95% CI (1.01, 2.96)] compared to those who didn't report/hardly reported an impact on daily life. No significant effects were found for gender, education, allergy testing, pulmonary diagnosis, or medication use and/or behavioural measures (Table 3). None of the factors in the model were significantly related to a lack of perceived need for additional information on managing SAR (“No” vs. “Not sure”).

**Table 3:** Multinomial logistic regression analysis of factors associated with self-reported perceived need for additional information on managing SAR symptoms, The Netherlands, 2024.

Factors	Comparison 1: “Yes” vs. “Not sure”		Comparison 2: “No” vs. “Not sure”	
	OR (95%CI)	*p*-value	OR (95%CI)	*p*-value
Gender				
Female	1		1	
Male	1.25 (0.84–1.87)	0.264	0.80 (0.55–1.15)	0.224
Age (years)				
<35	2.37 (1.33–4.24)	0.004	0.78 (0.49–1.23)	0.282
35–44	1.78 (1.00–3.18)	0.051	0.76 (0.49–1.19)	0.228
45–54	1.32 (0.72–2.39)	0.368	0.65 (0.42–1.03)	0.064
>=55	1		1	
Education level				
Lower or medium education	1		1	
Higher education	1.32 (0.83–2.10)	0.240	0.88 (0.61–1.28)	0.501
University education	1.37 (0.88–2.12)	0.162	1.18 (0.79–1.75)	0.418
Allergy test performed				
No	1		1	
Allergy confirmed	0.95 (0.66–1.37)	0.793	1.35 (0.99–1.85)	0.062
Pulmonary co-diagnosis				
None	1		1	
One or multiple (Asthma, COPD, etc.)	1.11 (0.72–1.69)	0.640	0.93 (0.64–1.35)	0.714
Impact on daily life				
Not at all/hardly	1		1	
Moderate extent	1.73 (1.01–2.96)	0.045	0.77 (0.54–1.08)	0.131
Great extent/very great extent	1.27 (0.83–1.93)	0.266	0.67 (0.41–1.10)	0.116
Medication use and/or behavioural measures				
None	1		1	
Behavioural measures only, no medication	1.55 (0.45–5.39)	0.492	1.55 (0.51–4.70)	0.436
Medication only, no behavioural measures	0.99 (0.43–2.26)	0.997	1.31 (0.62–2.77)	0.479
Both medication and behavioural measures	1.25 (0.55–2.87)	0.592	1.41 (0.67–3.0)	0.366

The multinomial logistic regression analyses associations between sociodemographic and clinical variables and self-reported need for additional information on managing SAR symptoms [outcome categories “yes”, “no” and “not sure” (reference category)]. The 95% confidence interval for the odds ratio reflects where the true odds ratio is likely to fall 95% of the time. A *p*-value of <0.05 was considered statistically significant.

### 3.5 Perceived possible contribution of real-time pollen information on managing SAR symptoms

Out of the 710 (73.9%) participants who thought that real-time pollen information could contribute to managing SAR symptoms or were not sure, the most commonly reported expected use of real-time pollen information was for confirmation of allergy symptoms (53.8%), followed closely by alleviation of allergy symptoms (51.0%) and adjusting medication (49.3%). In contrast, only 21.8% of respondents indicated they would use the information to prevent symptoms.

A multinomial logistic regression analysis (Table 4) was conducted to examine whether socio-demographic factors (age, gender, education) and SAR symptom (management) factors (allergy testing, pulmonary diagnosis, medication use, behavioural measures, impact on daily life) are associated with perceived possible contribution of real-time pollen information on managing SAR symptoms. The overall model was significant [*χ*^2^(26) = 83.3, *p* < 0.001]. However, as with perceived information needs, the model explained a relatively low proportion of variance (Nagelkerke *R*^2^ = 0.1). This suggests that additional factors, not included in this study, also relate to perceived contribution. Two factors were shown to be associated with perceived possible contribution of real-time pollen information. Male participants were more likely to report a possible contribution of real-time pollen information on managing SAR symptoms (“Yes” vs. “Not sure”) [OR = 1.81 95% CI (1.25, 2.61)], compared to female participants. Furthermore, participants with a university education were more likely to report a possible contribution of real-time pollen information on managing SAR symptoms (“Yes” vs. “Not sure”) [OR = 2.01 95% CI (1.33, 3.03)] compared to participants with a lower/medium level of education. Age, allergy testing, pulmonary co-diagnosis, impact on daily life, and medication use and/or behavioural measures were not significantly associated with a perceived possible contribution of real-time pollen information on managing SAR symptoms (Table 4). None of the factors in the model related significantly to a lack of perceived possible contribution of real-time pollen information on managing SAR (“No” vs. “Not sure”).

**Table 4:** Multinomial logistic regression of factors that are associated with the self-reported perceived possible contribution of real-time pollen information for managing SAR symptoms, The Netherlands, 2024.

Factors	Comparison 1: “Yes” vs. “Not sure”		Comparison 2: “No” vs. “Not sure”	
	OR (95%CI)	*p*-value	OR (95%CI)	*p*-value
Gender				
Female	1		1	
Male	1.81 (1.25–2.61)	0.002	1.24 (0.82–1.87)	0.303
Age (years)				
<35	1.00 (0.63–1.59)	0.993	0.82 (0.48–1.40)	0.467
35–44	0.70 (0.44–1.13)	0.148	1.34 (0.82–2.20)	0.249
45–54	0.82 (0.51–1.31)	0.399	0.85 (0.50–1.45)	0.551
>=55	1		1	
Education level				
Lower or medium education	1		1	
Higher education	1.32 (0.90–1.94)	0.155	0.86 (0.57–1.30)	0.484
University education	2.01 (1.33–3.03)	<0.001	1.41 (0.91–2.17)	0.128
Allergy test performed				
No	1		1	
Allergy confirmed	0.89 (0.65–1.23)	0.493	0.93 (0.66–1.32)	0.683
Pulmonary co-diagnosis				
None	1		1	
One or multiple (Asthma, COPD, etc.)	1.40 (0.97–2.02)	0.074	0.77 (0.50–1.19)	0.237
Impact on daily life				
Not at all/hardly	1		1	
Moderate extent	1.14 (0.79–1.64)	0.477	0.75 (0.52–1.10)	0.145
Great extent/very great extent	0.87 (0.53–1.42)	0.577	0.72 (0.43–1.23)	0.231
Medication use and/or behavioural measures				
None	1		1	
Behavioural measures only, no medication	1.36 (0.43–4.27)	0.599	0.41 (0.12–1.39)	0.151
Medication only, no behavioural measures	0.89 (0.38–2.08)	0.787	0.67 (0.31–1.43)	0.301
Both medication and behavioural measures	1.56 (0.67–3.63)	0.302	0.55 (0.25–1.19)	0.129

The multinomial logistic regression analyses associations between sociodemographic and clinical variables and self-reported perceived possible contribution of real-time pollen information for managing SAR symptoms [outcome categories “yes”, “no” and “not sure” (reference category)]. The 95% confidence interval for the odds ratio reflects where the true odds ratio is likely to fall 95% of the time. A *p*-value of <0.05 was considered statistically significant.

## 4 Discussion

This study provides a valuable first step in understanding whether and how providing real-time pollen data aligns with the needs of SAR patients. The study looked at current symptom management strategies, and factors related to information needs and the perceived potential for real-time pollen data to aid symptom management.

Nearly half of the respondents reported worsening, earlier onset or longer duration of symptoms in recent years. This aligns with evidence of climate-related changes in pollen concentrations and seasons (21, 22). However, these findings rely on retrospective self-reporting and may be subject to recall bias. Most participants used allergen avoidance measures and pharmacotherapy, yet perceived effectiveness varied. Over 15% reported limited relief, suggesting that additional measures might be beneficial and/or that timing and consistency in taking medication can be improved. Allergen avoidance and pharmacotherapy are most effective when initiated prior to exposure and then maintained throughout the allergy season (11, 13, 23).

The findings of this study resonate with a growing European consensus that the full potential of current SAR management strategies—focussing on avoidance, pharmacotherapy, and immunotherapy—has yet to be fully realized (1, 24). Individualized, real-time pollen information is increasingly recognized as a possibly valuable tool to enable more preventive and reactive symptom management (18). Nevertheless, real-time pollen information will only be effective in reducing symptom burden if SAR patients seek out and use this information. In this study, the majority of participants reported at least some need for additional information on symptom management and thought that real-time pollen information could (potentially) contribute to managing SAR symptoms. However, consistent with prior studies, participants primarily valued real-time pollen data for symptom confirmation, while its use for prevention purposes was less commonly mentioned (20). This underlines a persistent gap between the more frequently reported reactive behaviour and the proactive behaviour that leads to optimal effectiveness of medication, i.e., taking medication prior to exposure and the appearance of symptoms. Real-time pollen information may help bridge this gap by supporting earlier, exposure-informed decision-making. However, implementation requires not only technical availability but also alignment with end-user needs, digital skills, and educational support (17).

Younger age and higher impact on daily life were significantly associated with greater informational needs regarding symptom management. Perceived usefulness of real-time pollen data on managing SAR was associated with male gender and university education. These associations might be partly explained by literature indicating that health-literate individuals tend to be more proactive in managing environmental triggers (19, 25). However, the relatively low explanatory power of the models suggests that additional, unmeasured factors influence informational needs and perceived usefulness.

This study primarily focused on socio-demographic factors and symptom (management) factors. Future research could further examine relevant factors including psychological beliefs, such as beliefs about one's ability to manage symptoms effectively (i.e., self-efficacy), and beliefs around perceived costs and barriers to using real-time pollen information. We would also recommend further research into information seeking behaviours, building on current findings (26).

This study has several strengths, including the comprehensive assessment of symptom management, behaviours, and informational preferences. However, various limitations must be acknowledged. The coordinated recruitment approach through social media ensured broad visibility, but may have resulted in a self-selected sample of digitally engaged, health-aware individuals with more perceived SAR symptoms, limiting generalizability. Over half of participants reported SAR without clinical confirmation, which may reduce diagnostic accuracy; however, our outcomes focus on perceived needs and behaviours rather than clinical diagnosis. Potential misclassification may attenuate associations and limit external validity, but is unlikely to invalidate the observed patterns. The forced-response design may have introduced response bias for items perceived as less relevant but the inclusion of “not sure” response options probably minimized this effect. Finally, the cross-sectional design precludes causality.

In conclusion, this study shows unmet informational needs and a perceived potential for real-time pollen data to support symptom management among individuals with SAR in the Netherlands. Notably, the potential for earlier treatment highlights an opportunity for digital tools to facilitate earlier, exposure-informed self-management. However, this requires SAR patients to seek out and use these tools. Younger age and higher impact on daily life were associated with greater informational needs, and male gender and higher educational level were associated with higher perceived usefulness of real-time pollen data. These insights offer direction for targeted implementation. Future work should explore how to implement such tools in an accessible and equitable manner.

## Data Availability

The raw data supporting the conclusions of this article will be made available by the authors, without undue reservation.
